# Summary of the best evidence for non-pharmaceutical interventions for mild cognitive impairment in Parkinson’s disease

**DOI:** 10.3389/fneur.2025.1598974

**Published:** 2025-07-03

**Authors:** Yud Dan Liu, Hui Fang Li, Ya Xian Zhai, Yun Xia Shen, Jinmei Yang, Li Mei He, Ting Shen

**Affiliations:** ^1^School of Nursing, Yunnan University of Traditional Chinese Medicine, Kunming, China; ^2^Kunming Municipal Hospital of Traditional Chinese Medicine, Kunming, China

**Keywords:** Parkinson’s disease, mild cognitive impairment, non-pharmacological interventions, evidence summary, best evidence

## Abstract

**Objective:**

This study aimed to synthesize and evaluate the best evidence for non-pharmacological interventions targeting mild cognitive impairment in Parkinson’s disease (PD-MCI), thereby informing the development of cognitive management strategies for this population.

**Methods:**

A systematic search was conducted across multiple databases (e.g., UpToDate, BMJ Best Practice, and Cochrane Library) up to November 2024. Two researchers independently screened literature, assessed quality using standardized tools (AGREE II, JBI criteria), and graded evidence.

**Results:**

Thirteen studies were included (five guidelines, two clinical decisions, two systematic reviews, one meta-analysis, and three RCTs). Twenty evidence points were categorized into six themes: safety/efficacy, assessment, cognitive training, exercise, health education, and multi-strategy approaches.

**Conclusion:**

This study consolidates evidence supporting non-pharmacological interventions for PD-MCI, offering actionable recommendations for clinical practice to delay progression to Parkinson’s disease dementia (PDD).

## 1 Introduction

Mild cognitive impairment in Parkinson’s disease (PD-MCI) is a transitional state between normal cognition and Parkinson’s disease dementia (PDD) (1). While PD-MCI may revert to normal cognition, it remains a critical risk factor for PDD, with up to 85% of patients progressing within 20 years (1). PDD severely impacts patients’ quality of life and increases caregiver burden, underscoring the urgency of early intervention (2). Current pharmacological options for PD-MCI are limited and may exacerbate adverse effects; however, non-pharmacological interventions offer a safer alternative by mitigating drug-related risks and potentially delaying PDD onset (3). This study synthesizes evidence to guide clinicians in optimizing cognitive management strategies for PD-MCI.

## 2 Methods

This review adhered to the Preferred Reporting Items for Systematic Reviews and Meta-Analyses (PRISMA) guidelines to ensure transparent reporting.

### 2.1 Inclusion criteria

Using the PIPOST model from the Evidence-Based Nursing Center at Fudan University, we identified the specific evidence-based problem (4). Inclusion criteria included: (1) Population: patients diagnosed with PD-MCI.(2) Intervention: non-pharmacological approaches, including cognitive training, physical exercise, and health education.(3) Professionals implementing the evidence: doctors, nurses, and rehabilitation specialists caring for PD patients.(4) Outcome measures: Overall cognitive function scores, along with objective neuropsychological test results for all cognitive domains (memory, attention, working memory, executive function, language ability, and visual–spatial ability). (5) Settings for evidence application: hospitals, communities, and rehabilitation centers. (6) Types of evidence: including clinical decision support, practice recommendations, guidelines, evidence summaries, expert consensus, systematic reviews, and randomized controlled trials.

### 2.2 Exclusion criteria

Exclusion criteria included: (1) outdated or low-quality articles, (2) articles with incomplete information or those lacking full-text access, and (3) direct translations or guide interpretations of the articles.

### 2.3 Search strategy

The search was conducted from top to bottom according to the evidence resource “6S” evidence hierarchy model (5), systematic combing of the up-to-date database, BMJ Best Clinical Practice Database, International Guidelines Collaboration Network Platform, China Guide Network, the UK National Institute of Health and Clinical Optimization Database, the National Guide Database, Ontario, Canada, Scottish Medical School Guide Network, New Zealand Guide Collaboration, and the European Parkinson’s Disease Association Network. Further information search was conducted on the evidence related to the non-pharmacological intervention of PD-MCI in Weitong, Cochrane Library, PubMed, Embase, CINAHL, Web of Science, CNN, CNKI, CBM, Wanfang, Vip, and other databases. The time limit for retrieval will be until November 2024. In searching the guide network, the search terms included “Parkinson’s disease / cognitive impairment / mild cognitive impairment / non-pharmacological intervention / non-pharmacological treatment,” the English search included “Parkinson’s disease / cognitive impairment / mild cognitive impairment / non-pharmacological intervention / non-pharmacological therapy.” The Chinese database search strategy takes CNKI as an example, (theme: Parkinson’s disease + ‘Parkinson’s disease (pd)’ + Parkinson’s disease patients) AND (theme: mild cognitive impairment + mild cognitive impairment + “mild cognitive impairment (mci)’) AND (theme: guidelines + expert consensus 10 system evaluation + Meta analysis + evidence summary). The English database retrieval formula should be based on PubMed as an example, and the search formula is shown in Table 1.

**Table 1:** PubMed search strategy.

Step	Retrieval type
#1	(“parkinson disease”[Title/Abstract] OR “idiopathic parkinson s disease”[Title/Abstract] OR “lewy body parkinson s disease”[Title/Abstract] OR “parkinson s disease idiopathic”[Title/Abstract] OR “parkinson s disease lewy body”[Title/Abstract] OR “paralysis agitans”[Title/Abstract] OR “parkinson s disease”[Title/Abstract] OR “lewy body parkinson disease”[Title/Abstract] OR “primary parkinsonism”[Title/Abstract] OR “parkinsonism primary”[Title/Abstract] OR “parkinson disease idiopathic”[Title/Abstract])
#2	“cognitive dysfunction”[MeSH Terms] OR (“cognitive”[All Fields] AND “dysfunction”[All Fields]) OR “cognitive dysfunction”[All Fields] OR (“cognitive dysfunction”[MeSH Terms] OR (“cognitive”[All Fields] AND “dysfunction”[All Fields]) OR “cognitive dysfunction”[All Fields] OR (“cognitive”[All Fields] AND “disorder”[All Fields]) OR “cognitive disorder”[All Fields]) OR (“cognitive dysfunction”[MeSH Terms] OR (“cognitive”[All Fields] AND “dysfunction”[All Fields]) OR “cognitive dysfunction”[All Fields] OR (“cognitive”[All Fields] AND “impairments”[All Fields]) OR “cognitive impairments”[All Fields]) OR (“cognitive dysfunction”[MeSH Terms] OR (“cognitive”[All Fields] AND “dysfunction”[All Fields]) OR “cognitive dysfunction”[All Fields] OR (“mild”[All Fields] AND “cognitive”[All Fields] AND “impairment”[All Fields]) OR “mild cognitive impairment”[All Fields]) OR (“cognitive dysfunction”[MeSH Terms] OR (“cognitive”[All Fields] AND “dysfunction”[All Fields]) OR “cognitive dysfunction”[All Fields] OR (“cognitive”[All Fields] AND “impairment”[All Fields] AND “mild”[All Fields]) OR “cognitive impairment mild”[All Fields]))
#3	(“guideline”[Publication Type] OR “guidelines as topic”[MeSH Terms] OR “guideline”[All Fields] OR ((“expert”[All Fields] OR “expert s”[All Fields] OR “expertize”[All Fields] OR “experts”[All Fields]) AND (“consensual”[All Fields] OR “consensually”[All Fields] OR “consensus”[MeSH Terms] OR “consensus”[All Fields])) OR (“systematic review”[Publication Type] OR “systematic reviews as topic”[MeSH Terms] OR “systematic review”[All Fields]) OR (“meta analysis”[Publication Type] OR “meta analysis as topic”[MeSH Terms] OR “meta analysis”[All Fields]) OR ((“evidence”[All Fields] OR “evidences”[All Fields] OR “evident”[All Fields] OR “evidently”[All Fields]) AND (“summaries”[All Fields] OR “summary”[All Fields])) OR (“recommend”[All Fields] OR “recommendable”[All Fields] OR “recommendation”[All Fields] OR “recommendation s”[All Fields] OR “recommendations”[All Fields] OR “recommended”[All Fields] OR “recommending”[All Fields] OR “recommends”[All Fields]))
#4	#1 AND #2 AND #3

### 2.4 Literature quality evaluation standards

Based on the type of research literature, appropriate evaluation tools are selected for assessing the quality. The guidelines use the Appraisal of Guidelines for Research and Evaluation II (AGREE II) (6) to conduct quality evaluation. AGREE II includes 23 items across 6 domains, with each item scored on a scale of 1 to 7, where 1 indicates “strongly disagree” and 7 indicates “strongly agree.” The standardized percentage for each domain score is calculated as follows: [(total score of all assessors–minimum possible score) / (maximum possible score–minimum possible score)] × 100%. The minimum possible score for each domain is the number of items scored 1 point, and the maximum possible score is the number of items scored 7 points (7). A higher standardized percentage indicates better guideline quality. The guideline recommendation grade is classified into three levels: A for a standardized percentage > 60%, B for a standardized percentage between 30 and 60% in 3 or more domains, and C for a standardized percentage < 30% in more than 3 domains (8). The quality evaluation of expert consensus, systematic reviews, meta-analyses, and randomized controlled trials was conducted according to the Australian JBI (2016) guidelines, with each item assessed as “yes,” “no,” “unclear,” or “not applicable” (9).

### 2.5 Evidence summary

All members involved in the literature screening and quality evaluation underwent systematic training in evidence-based nursing science, and classified and summarized the evidence according to the theme.

## 3 Results

### 3.1 Literature search results and general characteristics of the included studies

**Figure 1:**
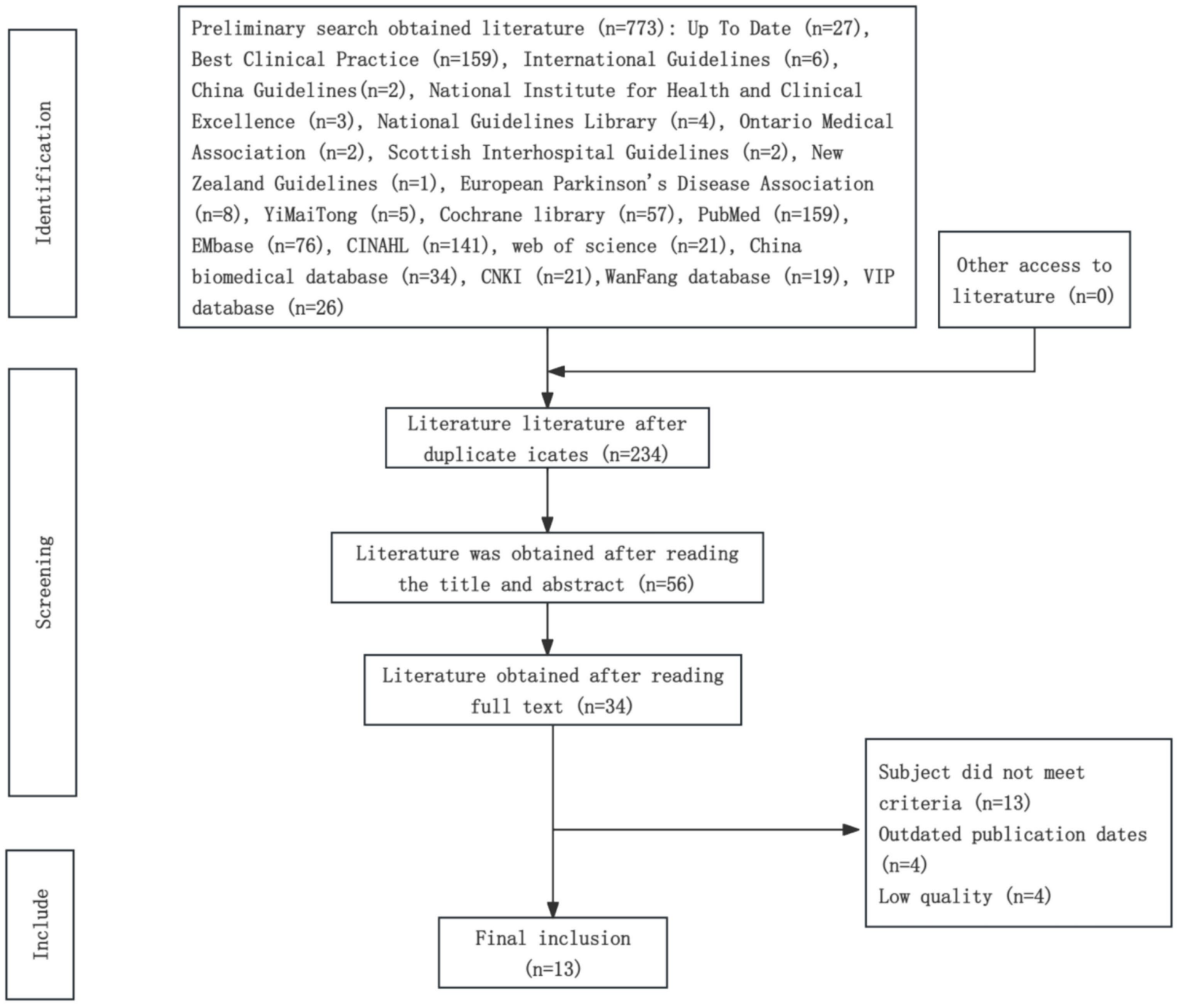
Flow chart of literature screening.

A total of 763 articles were retrieved and imported into NoteExpress for screening. After excluding duplicates, 234 articles remained. Following the title and abstract review, 56 articles were selected. After full-text reading, 34 articles were further shortlisted; 3 studies were excluded because their study populations did not meet the inclusion criteria, 4 studies were excluded due to outdated publication dates, and 3 studies were excluded because of low methodological quality. Consequently, 13 articles were included: 2 clinical decisions, 5 clinical guidelines, 2 systematic reviews, 1 meta-analysis, and 3 randomized controlled trials (RCTs). The literature screening flowchart is presented in Figure 1, and the general characteristics of the included studies are summarized in Table 2.

**Table 2:** General characteristics in the literature.

Number	Literature	Published time(Year)	Country	Literature reference	Type of literature	Literature theme
1	Elke Kalbe (3)	2024	Germany	PubMed	Guideline	Diagnosis of cognitive and affective disorders in patients with PD. A new focus of non-pharmacological interventions
2	L WANG (10)	2021	China	CNKI	Guideline	Guidelines for the diagnosis and treatment of PD-MCI in China
3	Tang Yi (26)	2022	China	CNKI	Guideline	Chinese Guidelines for Cognitive Training
4	Höglinger G (27)	2024	Germany	PubMed	Guideline	Diagnosis and treatment of PD
5	Grimes D (28)	2019	Canada	PubMed	Guideline	Management of PD
6	Pupíková M (29)	2020	Vienna	PubMed	clinical Decision	Non-pharmacological management of cognitive impairment in PD
7	Jennifer G Goldman (30)	2018	America	Up To Date	clinical Decision	Cognitive impairment and dementia in PD
8	Hanna M. Gavelin (31)	2022	Sweden	PubMed	Systemic review	Computerized cognitive training in PD
9	Wang Y (32)	2022	China	PubMed	Systemic review	Effect of Tai Chi and Qigong on cognition in neurological disorders
10	Lawrence BJ (33)	2017	Australia	PubMed	Meta-analysis	Parkinson’s disease cognitive training and non-invasive brain stimulation of cognition
11	Schmidt N (34)	2021	Germany	PubMed	RCT	Multi-domain group cognitive training enhances memory in patients with PD-MCI
12	Liu Z (35)	2020	China	CNKI	RCT	Transcranial repetitive needle stimulation for PD-MCI
13	Liao, Z (36)	2021	China	CNKI	RCT	Efficacy of high-frequency repetitive TMS on mild cognitive dysfunction in early PD

### 3.2 Quality evaluation results of the included studies

Five guidelines were included in this study. The AGREE II standardized scores for each domain are shown in Table 3. Four of the guidelines are recommended for grade B, while one is recommended for grade A. Overall, the quality of the guidelines is high. All of them were included. Two clinical decisions were included in this study. In terms of recommendations, both articles in the latest column were rated as “no,” while the rest were rated as “yes” or “partially yes.” Specific details are shown in Table 4. Overall, the quality is high, and all were included. Two systematic reviews, one meta-analysis, and three randomized controlled trials (RCTs) were included in this study. After evaluation using the criteria from the Australian JBI Evidence-Based Healthcare Centers, the item “reduce data extraction error measures” from Hanna M. Gavelin’s study was rated as “unclear.” The remaining literature and corresponding criteria were all rated as “yes.” The study designs were relatively complete, and the overall quality of the literature was high. All were included.

**Table 3:** Results of the methodological quality evaluation of the included guidelines.

Inclusion guidelines	Standardized percentage of scores across fields						Number of fields>60%	Number of fields <30%	Level recommendation
	Scope and purpose	Guidelines are developed for the participants	Develop Rigor	Clear statement and expression	Applicability	Compilation of independence			
Elke Kalbe (3)	63.9	75	45.3	62.5	82.3	70.8	5	0	B
Günter Höglinger G (27)	97.2	83.3	29.1	73.6	58.3	100	5	1	B
L WANG (10)	87.5	73.6	39.6	72.2	83.3	50	4	0	B
Tang Yi (26)	88.9	88.9	62.5	90.3	65.6	50	5	0	B
Grimes D (28)	94.4	91.7	92.7	94.4	85.4	91.7	6	0	A

**Table 4:** Results of the clinical decision methodology.

Included in the literature	Scope and application are specific	Authorship is transparent	Reader/ Edit is transparent	Whether the search method is transparent and comprehensive	Whether the evidence grading system is transparent and translatable	Whether the suggestion is clear	Whether the recommendation is properly cited	Whether the advice is up to date	Is the conclusion fair	Can this summary be applied to your patient
Jennifer G Goldman (30)	Yes	Yes	Yes	Part is	Part is	Yes	Yes	No	Yes	Yes
Pupíková M (29)	Yes	Yes	Yes	Part is	Part is	Yes	Yes	No	Yes	Yes

### 3.3 Summary of evidence

Due to significant heterogeneity in intervention types (e.g., cognitive training protocols and exercise modalities) and outcome measures (e.g., MoCA and MDRS), quantitative pooling was not feasible. Instead, evidence was synthesized thematically, with quality assessed using AGREE II and JBI tools to ensure rigor. In addition, differences in intervention design (e.g., duration and frequency) and cultural adaptation (e.g., the use of Tai Chi in Chinese studies) led to heterogeneity. We addressed this issue by dividing the evidence into six themes and prioritizing high-quality guidelines (AFREE II ≥ 60%) and randomized controlled trials. A total of 22 relevant pieces of evidence were extracted from the 13 included studies on PD-MCI patients. The evidence was organized into six main themes: safety and effectiveness, assessment and screening, cognitive intervention, exercise, rTMS, health education, and multi-strategy interventions. The details are provided in Table 5.

**Table 5:** Summary of the best evidence for non-pharmacological interventions in PD-MCI.

Evidence theme	Description of evidence
Safety and effectiveness	1.PD-MCI is a major risk factor for the development of PDD, which significantly reduces the quality of life in PD patients. It also increases the risk of nursing home placement, death, and caregiver burden. Non-pharmacological treatments for PD-MCI can enhance or stabilize cognitive function (3).
Assessment and Screening	A level I diagnosis of PD-MCI requires a MoCA score <26, a cutoff of <140 for MDRS or SCOPA-COG, and ACE or ACER values adjusted for education, with a PANDA cutoff of <17. For grade diagnosis, consider the following aspects (3): For Level II tests, use two tests for each of the five cognitive domains: executive function, attention/working memory, memory, language, and visual cognition. Use at least two different functions within each domain to diagnose cognitive impairment. Define cognitive impairment using values 1–2 standard deviations below the mean.
	3.A general cognitive function assessment scale is recommended for a brief evaluation of all PD patients. Medical units with clinical research needs should perform a comprehensive cognitive evaluation for PD-MCI patients, covering five cognitive domains: executive function, working memory and attention, visual–spatial function, language, and memory, using at least two scales for each domain (10).
	4.Depression screening is recommended as a potential cause or contributing factor to cognitive impairment (30).
Cognitive intervention	5.Cognitive interventions, such as cognitive training, repetition, and stimulation, are considered the most effective non-pharmacological methods, with cognitive training specifically recommended for patients with mild cognitive impairment (3).
	6.A computerized cognitive training (CCT) approach is recommended for intervention (31).
	7.Both standard and tailored cognitive training are recommended to improve executive function, attention, working memory, and memory in PD (32).
	8.Multi-domain cognitive training, including group tasks, activity games, personal exercises, homework, and psychoeducation, is an effective treatment for memory and executive function in PD-MCI (34).
	9.Information technology can be fully utilized through daily task lists or task logs to conduct real-time, cross-context, and online monitoring (26).
	10.Cognitive training should ensure appropriate intensity and adequate duration. It is recommended that each session last at least 30 min, occurs no fewer than three times a week, and the total continuous training time should be no less than 20 h (26).
Exercise training	11.Aerobic exercises, such as treadmill training, are recommended for patients with PD-MCI (10).
	12.Tango training may help improve visual–spatial function in PD patients (10).
	13.Tai Chi and Qigong are effective methods for improving cognitive function in PD-MCI (31).
	14.Aerobic physical training should be conducted 2–3 times per week for 45–60 min to treat PD-MCI (3).
	15.Physical endurance training in the aerobic range should be performed 2–3 times per week, with each session lasting 45–60 min (27).
Health education support	16.It is recommended to provide oral and written communication throughout the course of the disease, and this should be used to strengthen individualized health education based on patient needs (28).
	17.Patients are encouraged to change their lifestyle and actively participate in social activities (28).
Multi-strategy intervention	18.Transcranial repeated injection stimulation for PD-MCI can significantly improve the clinical symptoms and improve the quality of life of patients (34). High-frequency r-TMS can improve the memory ability of patients with early PD-MCI (36, 37).
	19.Qualified medical units may consider left DLPFC tDCS treatment or tDCS combined with cognitive training treatment in patients with PD-MCI (10).
	20.Dual-task training combining physical exercise and cognitive training is recommended (29).

## 4 Discussion

### 4.1 Early evaluation and intervention in PD-MCI patients to delay progression to PDD

In clinical practice, treatment of PD primarily targets alleviating motor symptoms. However, mild cognitive impairment (MCI) is insidious, often occurring early in the disease or even prior to motor symptoms, making it difficult to detect. If the disease progresses to PDD, it severely impacts patients’ ability to live independently and reduces their quality of life, while also increasing the caregiver burden. Evidence shows that PD-MCI is a major independent risk factor for progression to PDD. Diagnosis of PD-MCI can be made based on objective neuropsychological tests and clinical reports. Brief assessment methods can provide a preliminary evaluation of overall cognitive function; however, institutions with sufficient resources should use specific assessment scales to evaluate cognitive domain impairment and develop targeted interventions. Furthermore, studies have shown that PD patients with cognitive impairment often have co-occurring depression. Therefore, it is recommended to include depression screening in cognitive assessments to exclude risk factors and enable early intervention (10). Recent studies have suggested that objective physiological indicators, such as cerebrospinal fluid analysis, magnetic resonance imaging, and EEG, may be useful for diagnosing PD-MCI. These tests can be conducted based on patient preferences and the capabilities of medical institutions (11).

### 4.2 Develop effective cognitive intervention strategies to improve the cognitive function of PD-MCI

Cognitive intervention is increasingly recognized as the most effective and safest non-pharmacological strategy for PD-MCI and is gradually being tested and promoted. Cognitive interventions include cognitive training, cognitive rehabilitation, and cognitive stimulation. Among them, cognitive training, which includes traditional paper-based methods, computer-based training, and virtual reality-based training, is recommended for PD-MCI patients. Computer-based cognitive training is considered especially effective, as it integrates visual, auditory, and other sensory stimuli, which studies have shown improve focus and training outcomes (12). With the rise of big data and mobile health, cognitive training is likely to shift gradually from hospital-based to home-based rehabilitation. Cognitive training programs suitable for the elderly should be developed with the help of smartphones and other mobile devices. Additionally, big data should be used for dynamic evaluation and feedback to enhance the sustained effectiveness of cognitive intervention. Additionally, personalized cognitive training programs should be tailored to the specific symptoms of PD-MCI patients. These programs should offer diverse forms of training, promote social interaction, and ensure appropriate intensity and duration. Guidelines recommend at least 30 min per session, at least three times a week, with a total of no less than 20 h of continuous training.

### 4.3 Development of health education programs and lifestyle modifications to enhance cognitive function

Clinical evidence indicates that detrimental lifestyle factors, including smoking, sedentary behavior, and alcohol abuse, may increase the risk of cognitive decline in PD patients by 23–41% (13). Comorbidities such as sleep disorders, diabetes, and hypercholesterolemia have also been shown to significantly accelerate cognitive deterioration (14, 15). Targeting these modifiable risk factors, health education and lifestyle interventions have emerged as pivotal strategies to delay the progression of PD-MCI:(1) Stratified Health Education: Tailored to patients’ cognitive levels and educational backgrounds, we use visual teaching tools (infographics/instructional videos) for disease education, emphasizing the clinical manifestations of PD-MCI, modifiable risk factors, and the potential progression to PDD. Behavioral interventions (smoking cessation, alcohol restriction, and sleep regulation) have demonstrated efficacy in decelerating cognitive decline (16). (2) Structured Exercise: Physical exercise exerts neuroprotective effects by promoting neuronal proliferation and enhancing cerebral function, thereby improving cognitive performance in PD patients (17). Current evidence-based exercise modalities for PD-MCI include Tai Chi, aerobic training, tango dancing, and recumbent cycling. While Tai Chi—originating from China—has demonstrated cognitive benefits across diverse populations (e.g., Spain, France, and South Korea) in dementia rehabilitation (18–20), most non-Chinese trials remain limited in scale. Large-scale multinational randomized controlled trials are warranted to standardize protocols and validate outcomes across heterogeneous healthcare systems. As summarized in Table 6, exercise frequency guidelines for cognitive training, aerobic exercise, and endurance training are aligned with current clinical recommendations.(3) Precision Nutrition Intervention: Dietary factors play a critical role in PD pathogenesis and management. Both Mediterranean diets and low-carbohydrate diets have shown significant improvements in executive function and verbal fluency (21, 22). We recommend increased dietary fiber intake to prevent constipation and vitamin D supplementation (400–800 IU/day). Vitamin D deficiency correlates with accelerated cognitive decline, while its anti-inflammatory and antioxidant properties may mitigate dementia risk (23). (4) Synergistic Effects: Multimodal interventions exhibit synergistic benefits for cognitive enhancement. Health education establishes a cognitive framework for disease understanding, exercise enhances cerebral function through neuroprotective mechanisms, and precision nutrition delays decline through metabolic modulation. Collectively, multidimensional lifestyle interventions regulate neuroplasticity and metabolic homeostasis, offering a safe and effective non-pharmacological approach for PD-MCI management.

**Table 6:** Intervention time.

Intervention	Frequency	Duration/Session	Evidence Source
Cognitive training	≥3times/week	30–60 min	Tang Yi (26)
Aerobic Exercise	2–3times/week	40–60 min	Grimes D (28)
Endurance training	2–3tomes/week	45–60 min	Höglinger G (27)

### 4.4 Explore the multi-strategy intervention synergy

PD-MCI exhibits heterogeneous cognitive impairment subtypes (e.g., attention deficit, executive dysfunction, and memory impairment) and significant interindividual variability in comorbid symptoms (e.g., depression and sleep disorders). Monotherapy approaches often fail to address these multifaceted needs, necessitating personalized intervention plans tailored to individual clinical profiles (24). Repetitive transcranial magnetic stimulation (rTMS), a non-invasive neuromodulation technique, has been investigated for addressing cognitive deficits in PD. In PD patients, rTMS protocols demonstrate therapeutic potential not only for motor symptom improvement but also for alleviating non-motor symptoms, including depression and cognitive dysfunction (24). Dual-task training, which requires simultaneous performance of two distinct tasks, enhances attentional control, processing speed, and cognitive flexibility. For PD patients, this approach can be adapted to functional scenarios such as ambulation, stair negotiation, and dressing. Ecologically relevant activities—including simulated driving and shopping tasks—not only improve cognitive performance but also enhance gait stability, balance control, and self-efficacy during daily activities, thereby effectively reducing fall risk (25). Future research should focus on developing precision intervention frameworks that integrate individualized cognitive profiles, neurophysiological biomarkers, and real-world functional demands to optimize therapeutic synergy in PD-MCI management.

### 4.5 Limitations and future research directions

However, studies on its effect on overall cognitive dysfunction remain inconclusive. Although some studies suggest that repeated rTMS may benefit cognitive function in PD patients, its effects remain unclear. Therapeutic targets and parameters are inconsistent, and further clinical studies are needed for confirmation. While Chinese studies contributed valuable insights (e.g., Tai Chi and transcranial needle stimulation), regional differences must be acknowledged. China’s healthcare system often integrates traditional practices into rehabilitation, which may not be directly applicable in Western settings. Additionally, cultural acceptance of home-based training and family-led care differs from individual-centric models elsewhere. Demographic factors (e.g., older age at diagnosis in Chinese cohorts) may also influence outcomes. Future multinational trials are needed to validate these interventions across diverse populations. While cognitive scores remain primary endpoints, patient-reported outcomes (e.g., daily functioning) are equally vital. Health education improved self-management, yet no trials have assessed long-term social participation. Future studies should adopt holistic metrics such as the WHOQOL-BREF. Based on the aforementioned research findings, a dual-track model of “disciplinary collaboration + intelligent monitoring” can be established in the future. On one hand, this involves developing multidisciplinary teams in neurology, rehabilitation, nutrition, and psychology to formulate comprehensive plans that include medication guidance, physical function exercises, and health education. On the other hand, it strengthens family and community support by using smart bracelets, APPs, and other devices to monitor patients’ activities and cognitive states in real-time, providing remote training guidance, and regularly revising the plan through follow-up visits. This integrated management system of “hospital-community-family” is expected to break through the time and space limitations of traditional interventions, achieving precise and continuous rehabilitation for PD-MCI patients.

## 5 Summary

This study summarizes 20 key pieces of evidence for non-pharmacological interventions in PD-MCI patients, covering safety, effectiveness, assessment, cognitive intervention, exercise, health education, and multi-strategy approaches, providing a relevant basis for clinical staff to implement these interventions. However, due to the inclusion of both domestic and international research with high heterogeneity between interventions, standardized practice recommendations cannot be made. In the future, healthcare professionals should continuously enhance their knowledge and skills in managing cognitive function in PD-MCI patients and develop diverse non-pharmacological intervention programs through multidisciplinary teamwork, offering strategies for cognitive rehabilitation from hospital to home. This will help improve the cognitive function of PD-MCI patients, delay progression to PDD, and enhance patients’ quality of life.

## Data Availability

The original contributions presented in the study are included in the article/supplementary material, further inquiries can be directed to the corresponding author.
